# Detection of safety helmet and mask wearing using improved YOLOv5s

**DOI:** 10.1038/s41598-023-48943-3

**Published:** 2023-12-05

**Authors:** Shuangyuan Li, Yanchang Lv, Xiangyang Liu, Mengfan Li

**Affiliations:** 1 Information Construction Office, Jilin Institute of Chemical Technology, Jilin City, 132022 China; 2 College of Information and Control Engineering, Jilin Institute of Chemical Technology, Jilin City, 132022 China

**Keywords:** Engineering, Mathematics and computing

## Abstract

With the advancement of society, ensuring the safety of personnel involved in municipal construction projects, particularly in the context of pandemic control measures, has become a matter of utmost importance. This paper introduces a security measure for municipal engineering, combining deep learning with object detection technology. It proposes a lightweight artificial intelligence (AI) detection method capable of simultaneously identifying individuals wearing masks and safety helmets. The method primarily incorporates the ShuffleNetv2 feature extraction mechanism within the framework of the YOLOv5s network to reduce computational overhead. Additionally, it employs the ECA attention mechanism and optimized loss functions to generate feature maps with more comprehensive information, thereby enhancing the precision of target detection. Experimental results indicate that this algorithm improves the mean average precision (mAP) value by 4.3%. Furthermore, it reduces parameter and computational loads by 54.8% and 53.8%, respectively, effectively striking a balance between lightweight operation and precision. This study serves as a valuable reference for research pertaining to lightweight target detection in the realm of municipal construction safety measures.

## Introduction

The rapid economic growth has propelled increased focus on municipal engineering development, particularly in urban areas. Unlike other engineering projects, municipal engineering involves prolonged durations, diverse outdoor activities, and is influenced by factors like concurrent operations, traffic, climate, and environment. This uniqueness poses distinct challenges to safety management. Construction tasks in public spaces demand adherence to safety protocols, including mandatory helmets and protective gear. However, lapses in safety awareness or supervision can result in unsafe behaviors^1^. Moreover, the risk of infectious diseases, such as influenza, underscores the importance of robust safety measures, especially regarding mask-wearing on construction sites. Overall, the occurrence of safety incidents and disease transmission emphasizes the need for improved safety management and self-protection measures in municipal engineering^2,3^.

Conventional manual supervision, while essential, can be tedious and prone to oversight. Supervisors may inadvertently miss critical details, resulting in insufficient oversight and a hidden increase in safety risks. Therefore, the implementation of intelligent monitoring systems capable of automatically identifying safety hazards holds significant importance for ensuring the successful completion of projects^4^.

Intelligent monitoring, which integrates deep learning with target detection technology, offers several advantages, due to the powerful pattern recognition and prediction ability of deep learning^5^. It enhances the efficiency of personnel safety inspections, reduces the need for a large safety management staff on construction sites, and effectively reduces construction supervision costs. Real-time monitoring through intelligent systems enables prompt issue identification and the formulation of corresponding measures. Moreover, intelligent monitoring promptly detects unsafe behaviors, such as failure to wear helmets or masks, facilitating accurate communication of corrective actions and the effective implementation of construction safety management. Intelligent monitoring elevates both detection accuracy and operational effectiveness while enhancing the efficiency of enterprise safety management. Not only does it safeguard the lives and assets of construction workers, but it also elevates the standard of construction safety management, ensuring that projects are completed on schedule^6^.

To mitigate overfitting, this experiment employs the Mosaic + Mixup data augmentation method to enhance the self-made dataset. The choice of this method for data augmentation over generative adversarial networks (GANs) is driven by its simpler, more efficient approach and greater training stability, effectively bypassing the complexities associated with GANs^7^. The YOLOv5s network structure is upgraded by replacing the initial YOLOv5s backbone network with a lightweight convolutional neural network, ShuffleNetv2. The ECA attention mechanism is employed to minimize the impact of dimensionality reduction on channel attention learning. Additionally, the EIoU loss function is optimized to address the issue of a poorly converging initial loss function, thereby improving accuracy. The algorithm presented in this paper effectively addresses the limitation of single-target detection and achieves simultaneous detection of multiple targets. Furthermore, it enhances real-time detection speed and the ability to detect obstructed targets, and it can better realize the detection of whether the workers are wearing safety helmets and masks at the same time, which provides technical support for the future development of municipal engineering safety.

The remainder of this paper is structured as follows: the second section provides an overview of the current research landscape both domestically and internationally. The third section offers a concise introduction to several leading deep learning algorithms, elucidates the YOLOv5s algorithm, and provides a detailed account of the three enhancement measures employed in this study. The fourth section delves into the composition of the dataset and the choice of the training platform. In the fifth section, the experimental process and results are presented, and finally, the sixth section serves as a comprehensive summary of this paper.

## Related work

First, with the rapid advancement of artificial intelligence technology, numerous experts have conducted extensive research in the field of mask recognition, proposing effective algorithms. Wei et al. introduced an enhanced network model called "Face_mask Net" based on YOLOv3 for mask detection. This improvement aimed to address shortcomings in the YOLOv3 model, where detection rates for small targets were low, it could not determine if the prediction frame intersected with the target frame when IoU values were identical, and traditional NMS often led to false suppression due to occlusion. The "Face_mask Net" enhanced residual blocks and the neural network structure, introduced SPP and CSPNet network modules, and employed DIoU as the loss function and DIoU-NMS algorithm as the classifier, significantly boosting target detection precision^8^. Cheng et al. proposed an enhanced YOLOv4-tiny mask detection method to aid in collaborative pandemic control. This approach addressed the challenges of poor real-time performance and complex deployment in large crowds. Within this algorithm, based on YOLOv4-tiny, the CSP module was replaced with two Resblock-D modules to reduce feature extraction network complexity and enhance detection speed. The introduction of SPP expanded the network's receptive field, allowing it to accept input images of various sizes and improving algorithm robustness. Additionally, a two-layer CA attention mechanism was incorporated to enhance algorithm utilization and detection accuracy^9^. Xiao et al. introduced a mask recognition method using a YOLOV5 model, primarily optimizing and improving initial model convolutional layer modules^10^. Liu et al. improved the Faster R-CNN algorithm by redesigning anchor sizes through K-means, enhancing detection in crowded spaces^11^. Shylaja et al. followed the principle of transfer learning, incorporating pre-training weights into model training. They conducted experiments on two mask face datasets with ordinary and complex backgrounds, achieving an average precision of 98.5%^12^. Furthermore, experts and scholars in related fields have studied the technology of automatic helmet identification. Shi et al. employed Image Pyramid to extract feature maps of various scales and detect helmet wearing in conjunction with YOLOv3^13^. Xie et al. introduced an enhanced YOLOv4 helmet detection algorithm (SMD-YOLOv4), which allowed for the acquisition of more target features in extremely complex backgrounds without compromising network inference speed^14^. Song et al. incorporated the CoordAtt coordinate attention mechanism into the YOLOv5s network backbone to allocate more attention to helmets and enhance the detection of small targets. They also replaced the residual block in the backbone network with the Res2NetBlock structure to address fusion deficiencies in the original backbone and enhance YOLOv5s' capability to fuse fine-grained information^15^. Zhang et al. conducted research on safety helmet detection based on deep learning, employing the Tensorflow framework and the Faster R-CNN method for real-time detection, yielding results with an average precision exceeding 90%^16^. Espinosa et al. utilized the EspinosaNetv2 model to enhance the convolutional extraction process in Faster-RCNN. This resulted in a simplified convolutional network with six layers (four convolutions), reducing parameter load and achieving an average detection precision of 88.8% even with low-angle, moving camera shots and some occlusion^17^.

In summary, progress has been made in the independent recognition of masks and helmets. However, there has not been in-depth research in the engineering field, where challenges include the detection of overlapped targets and small objects, often leading to missed or erroneous detections. Given the current research landscape, this paper investigates a method for simultaneous detection and identification of masks and safety helmets, with an emphasis on enhancing detection speed and precision to further safeguard the personal safety of construction personnel.

### Methodology

Two primary methods exist in deep learning for object detection: one-stage and two-stage object detection algorithms^18^. The latter involves two distinct steps. Given that a significant portion of target objects may go undetected in an image, the first step involves identifying the approximate area of the detection object within the image, creating a sample frame. Subsequently, a convolutional neural network is employed to classify and locate the target within this area. Conversely, the former method does not require such intricate operations, offering a straightforward inspection process that delivers results by inputting the image to be assessed^19^.

Before the advent of the YOLO series of algorithms, the R-CNN series was widely employed. Utilizing the CNN approach for target detection, R-CNN initially generates candidate regions using a selective search algorithm. It then extracts features with CNN and classifies them using the SVM classifier. Finally, it locates the target via a regression model^20^. Addressing the issues of computational complexity and high memory usage, Fast R-CNN was introduced. Similar to its predecessor, it generates candidate regions using a selective search algorithm and feeds images into the VGG16 network for convolution operations. Notably, convolution is performed not for each region proposal but directly on the entire image, reducing redundant calculations^21^. Building on these developments, Faster R-CNN achieved further advancements, significantly enhancing detection speed and overall performance. A key enhancement was the introduction of RPN networks to generate candidate regions. All of the aforementioned algorithms necessitate the extraction of body regions followed by classification and identification, resulting in slower detection and complex model structures^22^. In contrast, one-stage target detection omits these steps, resulting in faster detection and simpler models^23^.

Joseph Redmon et al. introduced the one-stage target detection algorithm YOLOv1 in 2016. Its core concept is to transform the detection problem into a regression problem, which can be accomplished using a CNN network. Initially, input images are standardized, features are extracted through convolution, and classification and regression results are output via the fully connected layer^24^. YOLOv2, an improvement over YOLOv1, achieved higher precision through a series of optimization methods. YOLOv2 eliminated Dropout and incorporated Batch Normalization after convolution. It also employed high-resolution images for training and employed K-means clustering for anchor box template calculation during training^25^. YOLOv3 abandoned pooling and fully connected layers, relying solely on convolution. It implemented multi-scale prediction using feature pyramid networks (FPN) to enhance spatial representation within the network. Additionally, it replaced Darknet19 with Darknet53 to improve data characterization capability. Notably, YOLOv2's softmax loss was substituted with logistic loss to enhance detection precision stability^26,27^.

YOLOv4 optimized computational complexity by replacing Darknet-53 with CSPDarknet-53. Mosaic data augmentation and self-adversarial training (SAT) adversarial training were introduced to enhance network generalization and robustness. The FPN + PAN structure was employed as the Neck part of YOLOv4 to incorporate all feature maps in final classification^28^. Following these optimizations, YOLOv5 introduced three types of data augmentation at the input. It also employed preset anchor frames that adaptively calculated anchor frame values during training, enhancing detection precision^29^. In terms of feature extraction, YOLOv5 utilized the Focus operation to reduce the model's floating-point operations (FLOPs), thereby enhancing detection speed^30^.

**Figure 1:**
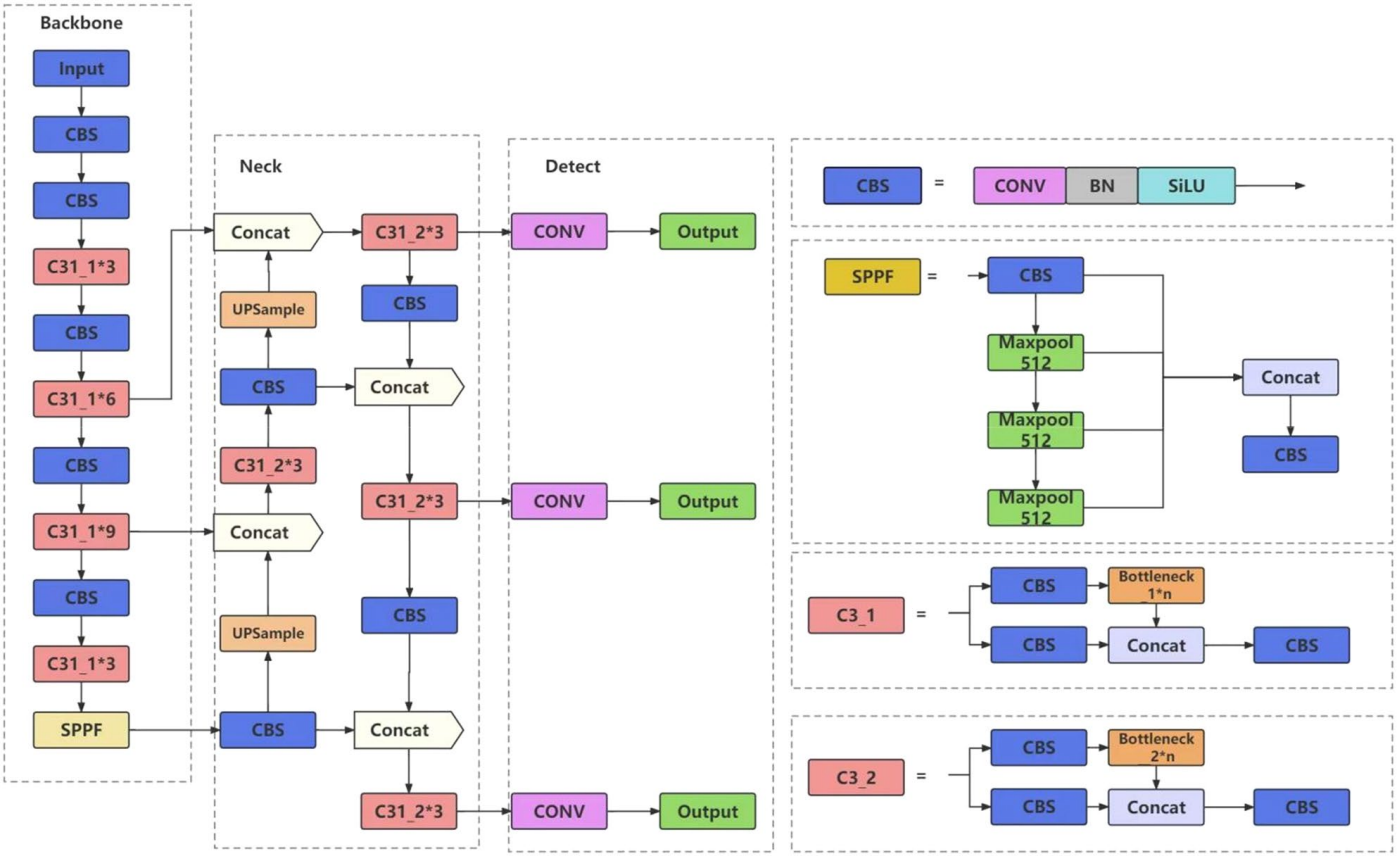
Network structure of YOLOv5.

The YOLOv5 network model comprises four versions: YOLOv5s, YOLOv5m, YOLOv5l, and YOLOv5x^31^. Based on existing research, this paper employs the YOLOv5s version, which meets the requirements for lightweight and real-time monitoring. YOLOv5s' weight data file is only 1/9th the size of YOLOv4's, allowing for an image inference speed of up to 0.007 s, which translates to processing 140 frames per second. This meets the demands for real-time video image detection, making YOLOv5s the chosen model for this paper. The model's structure consists of four parts: input, backbone, neck, and prediction. Refer to Fig. 1 for the YOLOv5s structure.

The input section comprises three modules: mosaic data enhancement, adaptive anchor box calculation, and adaptive image scaling. In the first module, a training image is selected, followed by the random selection of three images for operations such as random cropping, scaling, rotation, and translation. These images are then stitched together to create new training data. This process significantly enhances sample diversity and reduces the model learning complexity. The second module involves incorporating anchor frame calculations into the training process. During training, the predicted frame is output based on preset anchor frames, compared with the actual frame to obtain the offset between them, and then updated in reverse to adaptively determine the optimal anchor box values within the training set^32^. Self-adaptive image scaling automatically calculates the fill ratio for images of varying sizes encountered in real projects, thereby reducing the computational load on the model.

The backbone comprises a Focus structure and a CSP structure. The former slices the image before introducing it to the backbone^33^. The CSP structure divides the initial input into two channels: one directly undergoes convolution operations, while the other goes through multiple residual structures before convolution. Finally, the two channels are concatenated, enabling the model to learn more features.

The Neck section includes the FPN + PAN structure. FPN (feature pyramid network) employs a top-down approach to transfer high-level information to lower layers through upsampling, enhancing the entire pyramid. PAN, on the other hand, employs a bottom-up approach, complementing FPN by adding an inverted pyramid and transferring distinctive features from lower layers to upper layers, ensuring maximum retention of target features after combination^34^.

The prediction section employs GIOU_LOSS as the loss function in YOLO V5. Initially, the loss function struggled to determine if the predicted frame and real frame intersected or provide specific position information when they did^35^. GIOU_LOSS effectively addresses these issues.

## Structural adjustment of the deep learning algorithm

### Introduction of the lightweight convolutional neural network shuffleNetv2

The traditional YOLOv5s is burdened with numerous parameters and a large volume, making it challenging to deploy on terminal equipment. Additionally, it often suffers from loss of small object details during feature extraction^36^. Consequently, researchers have proposed various lightweight improvements. In this paper, we replace the YOLOv5s backbone network with the lightweight convolutional neural network ShuffleNetv2.

**Figure 2:**
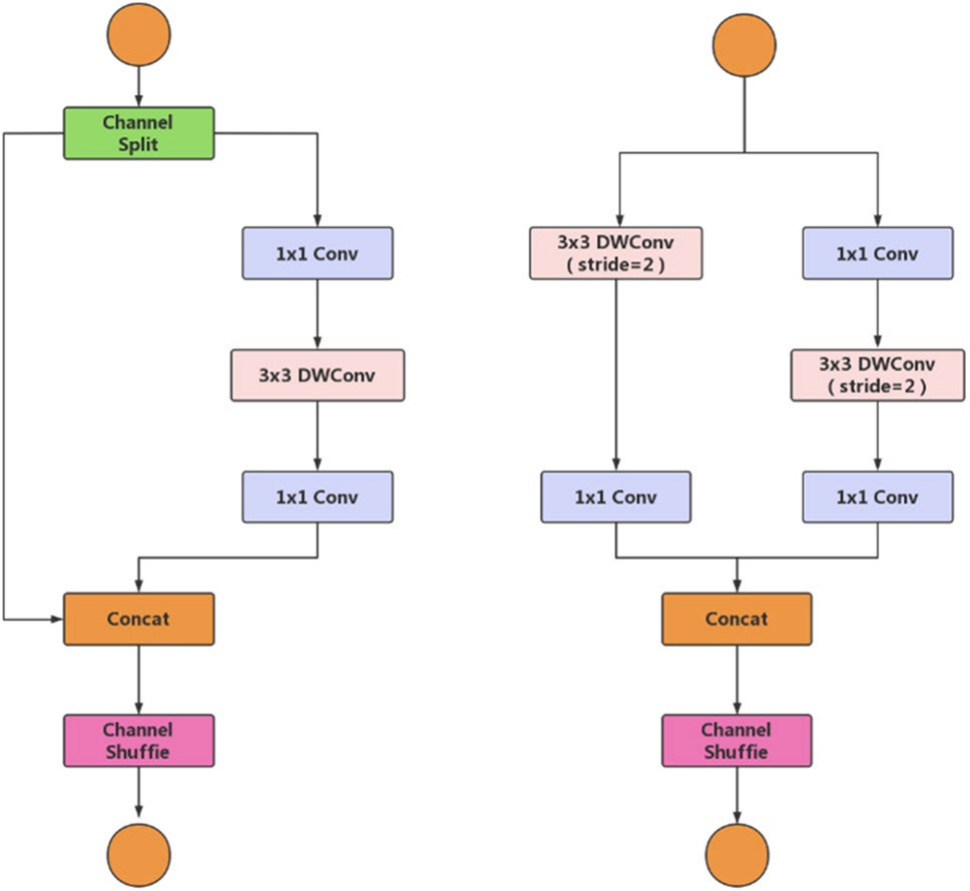
Basic module of ShuffleNetv2.

The ShuffleNetv2 block, based on the ShuffleNetv1 block, introduces the Channel Split operator. At the start of each block, the input feature map's c channels are split into two branches: c–c′ channels and c′ channels^37^. The left branch remains unchanged (shortcut connection), while the right branch performs a sequence of operations: a 1*1 convolution, followed by a 3*3 depthwise separable convolution, and finally another 1*1 convolution^38^. The results from the left and right branches are concatenated, resulting in the same number of channels as in the initial input. Subsequently, channel shuffle is performed on the concatenated output to promote information exchange between the left and right branches. In the ShuffleNetv2 module with a stride of 2, channel splitting is omitted, and one input is directly copied to each branch, granting each branch a stride = 2 downsampling. This halves the feature map's spatial size after Concat but doubles the number of channels, effectively completing downsampling^39^. Refer to Fig. 2 for a detailed illustration of this process.

### Integration of the ECA attention mechanism

The original YOLOv5s lacks a preference for attention when extracting target features^40^. Therefore, this paper introduces an attention module to address this limitation. The SE attention mechanism module, currently a mature module, initially conducts channel compression on the input feature map. However, the dimensionality reduction operation utilized introduces an unfavorable factor in channel attention, making the acquisition of channel relations inefficient and unnecessary^41^.

To address the aforementioned challenges, this paper introduces an enhanced version of the efficient channel attention (ECA) module, building upon the existing squeeze-and-excitation (SE) mechanism. In this augmentation, a 1*1 convolution is strategically employed to substitute the traditional fully connected layer (FC) within SE for the purpose of channel information acquisition. This modification proves to be instrumental in preventing the undesired reduction of channel dimensionality during the learning phase of channel attention information^42^. Consequently, this not only mitigates the risk of information loss but also leads to a reduction in the overall parameter volume involved in the learning process. This innovative approach enhances the model's capacity to effectively capture and leverage channel-specific information, thereby improving its performance and efficiency. The steps are outlined as follows:

- Input a feature map with dimensions H*W*C;
- Perform a global average pooling operation on the input feature map;
- Conduct channel feature learning on the compressed feature map, involving the acquisition of channel attention information via 1*1 convolution;
- Combine the obtained channel attention information with the original input feature map.

**Figure 3:**
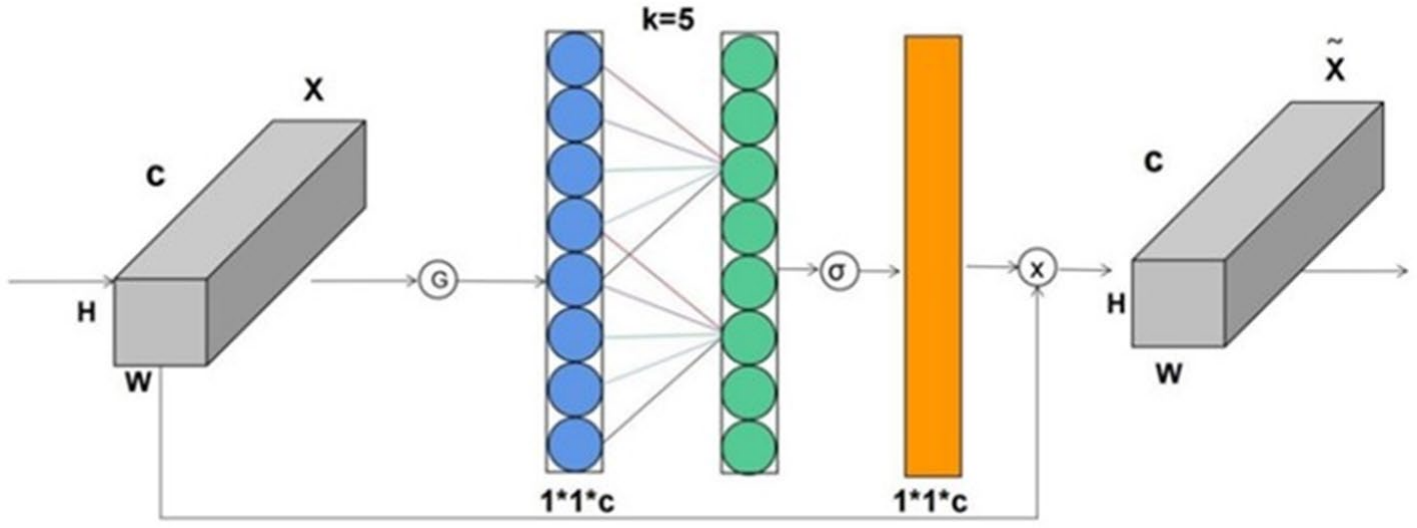
Flowchart of ECA attention mechanism.

In summary, the ECA attention mechanism offers a simpler operation compared to other attention mechanisms^43^. Moreover, it has minimal impact on network processing speed, aligning with the requirements for a lightweight and fast detection algorithm model in this paper^44^. The flowchart illustrating the ECA attention mechanism is presented in Fig. 3.

### Modification of the loss function

The loss function in YOLOV5s comprises three components: position, confidence, and category loss. The Intersection over Union (IoU) measures the ratio of the intersection area to the union area between the predicted boundary and the target boundary. A higher IoU value indicates a closer alignment between the prediction box and the target box, as denoted by Formulas (1) and (2).

1 $$\mathrm{IoU}=\frac{\mathrm{A}\cap \mathrm{B}}{\mathrm{A}\cup \mathrm{B}}$$

2 $$\mathrm{IoU}\text{Loss = 1}-\mathrm{IoU}$$

While IoU is a commonly used metric in target detection, it does not precisely capture the degree of overlap between two boxes^45^. YOLOv5s employs the generalized IoU (GIoU) Loss as the loss function to effectively address cases where bounding boxes do not overlap. However, GIoU has limitations; when the prediction box is entirely contained within the target box, GIoU degenerates into IoU, failing to evaluate the regression effect. To tackle this issue, the academic community has proposed improved loss function methods, such as complete IoU (CIoU) and distance IoU (DIoU). CIoU, however, faces challenges related to simultaneous width and height adjustments, and it does not consider the balance between easy and difficult samples^46^. While DIoU directly minimizes the acceleration convergence of the center point distance between the prediction box and the real box, it overlooks another crucial factor in bounding box regression^47^.

In this paper, we opt for the efficient IoU (EIoU) as the loss function. It introduces the focal loss while retaining the advantages of CIoU to address the sample imbalance issue in bounding box regression. Specifically, it reduces the optimal contribution of numerous anchor boxes with minimal overlap with the target box to the BBox regression. This ensures that the regression process focuses on high-quality anchor boxes^48^. The penalty term calculation formula is presented in Formula (3).

3 $${L}_{EIoU}={L}_{IoU}+{L}_{dis}+{L}_{asp}=1-IOU+\frac{{\rho }^{2}\left(b,{b}^{gt}\right)}{{c}^{2}}+\frac{{\rho }^{2}\left(\omega ,{\omega }^{gt}\right)}{{c}_{\omega }^{2}}+\frac{{\rho }^{2}\left(h,{h}^{gt}\right)}{{c}_{h}^{2}}$$

where ${c}_{\omega }$ and ${c}_{h}$ represent the width and height of the smallest bounding box covering both boxes, and *ρ* represents the Euclidean distance between b and a. The variables $\omega$, $h$, ${\omega }^{gt}$, and ${h}^{gt}$ denote the width and height of the predicted box and the actual box, respectively.

## Experimental setup

### Data set construction

The experimental data in this study pertain to the simultaneous detection of helmets and masks. Given the absence of a publicly available dataset, this paper creates a dedicated dataset through data collection, screening, and processing.

### Data collection

The dataset is compiled from video monitoring data obtained from construction sites and images collected from the internet. Frames extracted from construction site video monitoring or sourced from the internet are often considered background images, as they typically lack construction personnel, rendering them irrelevant to this study's research objectives. Consequently, these background images are identified and removed from consideration^49^. This paper initially screens the collected image data and selects pictures that meet the specified criteria as part of the annotation dataset, which is then divided into a training set and a test set, distributed randomly in an 80:20 ratio^50^. The training set and test set consist of 4000 and 1000 images, respectively.

### Data screening and processing

The data undergo preprocessing, and pictures that meet the criteria are converted into .jpg format. The model's robustness is enhanced by augmenting a portion of the positive sample data through operations like flipping and adjusting saturation and exposure^51^. All acquired data is manually labeled using the labeling tool labelImg, with construction personnel in the images categorized into four classes: wearing a helmet, wearing a mask, not wearing a helmet, and not wearing a mask. Subsequently, the images are processed into corresponding XML label files.

### Data augmentation technique

To alleviate the problem of overfitting and enhance the model's generalization performance, this paper employs a novel data augmentation approach. Building upon the Mosaic augmentation method, Mixup data augmentation is utilized. This involves combining images processed through Mosaic, feeding them to the network for training, and effectively enhancing the network's detection accuracy. Simultaneously, it enables the model to adapt better to complex real-world scenarios, thereby improving its generalization performance and robustness.

Mixup involves blending two images in a certain proportion to generate a new image. Subsequently, this new image and its corresponding labels are incorporated into the training process, as illustrated in the diagram below.

## Overview of the training platform

### Experimental environment

In this study, the experimental environment utilizes the Ubuntu 18.04 operating system, with programming carried out in Python. Model development, training, and result testing are all conducted within the PyTorch framework, leveraging the CUDA (compute unified device architecture) parallel computing architecture^52^. The configuration details are outlined in Table 1.

**Table 1:** Experimental configuration.

Experimental configuration	Specification
Operating system	Ubuntu18.04
Development language	Python3.9.2
Deep learning frame	Pytorch1.8.0
CPU	Intel Xeon
GPU	NVIDIA 2080TI
CUDA	CUDA11.2
IDE	PyCharm

### Network training

During the training of the YOLOv5s model, it is desirable to minimize the loss value within the model's loss function, with the ideal target being a value of 0. To optimize model performance, this study defines specific hyperparameters during training, as presented in Table 2.

**Table 2:** Network training hyperparameters.

Name of the training parameter	Parameter value
Initial learning rate	0.01
Weight attenuation factor	0.0005
Momentum	0.937
Batch	16
Epoch	300

## Results and discussions

### Evaluation metrics

This study employs the test set to assess the model's performance. When comparing it with the unoptimized YOLOv5s model, the evaluation of model performance relies on precision (P), recall (R), and mean average precision (mAP) as relevant indicators. Precision (P) measures the accuracy of algorithm-predicted results^53^, while recall (R) assesses the detection of complete targets using a detection algorithm^54,55^. The calculation formulas for P and R are presented in Formulas (4) and (5).

4 $$P=\frac{TP}{TP+FP}=\frac{TP}{alldetections}$$

5 $$R=\frac{TP}{TP+FN}=\frac{TP}{allgroundtruths}$$

In the provided formulas, TP represents the positive samples correctly identified by the model, FP denotes the positive samples incorrectly identified by the model, and FN indicates the targets not recognized as positive samples by the model. When calculating single-category precision (AP), the precision-recall curve and the area enclosed by the coordinate axes are determined using the integral method^56^. The mean average precision (mAP) is derived as the average of AP values across all categories. The calculation formulas are expressed in Formulas (6) and (7).

6 $$AP={\int }_{0}^{1}PdR$$

7 $$mAP=\frac{{\sum }_{i=1}^{N}A{P}_{i}}{N}$$

### Ablation experiment

In this paper, ablation experiments have been designed to assess the impact of each improvement strategy on the model's performance^57^. In these experiments, three enhancement measures, namely, the lightweight convolutional neural network ShuffleNetv2, the ECA attention module, and the EIoU loss function, are individually incorporated into the YOLOv5s algorithm model, and they are trained under identical experimental conditions. The training results are presented in Table 3, with a checkmark (√) indicating the employed improvement strategy.

**Table 3:** Comparison of ablation experiment results.

Algorithm model	ShuffleNetv2	ECA	EIoU	mAP@0.5	Parameter volume/M	Computing volume/M	Speed/s
YOLOv5s	–	–	–	0.832	7.05	14.5	0.0086
YOLOv5s-1	√	–	–	0.805	3.12	6.2	0.0074
YOLOv5s-2	√	√	–	0.825	3.16	6.4	0.0082
YOLOv5s-3	√	√	√	0.875	3.19	6.7	0.0096

The table above displays that the mAP (mean average precision) value of the original YOLOv5s algorithm model can achieve 83.2%. YOLOv5s-1 involves the introduction of the lightweight network structure ShuffleNetv2 into the YOLOv5s algorithm model, resulting in a 2.7% reduction in mAP value. However, this adjustment significantly reduces both parameter volume (55.8% reduction) and computational workload (57.3% reduction), albeit with a slight reduction in detection speed. YOLOv5s-2 introduces the attention mechanism ECA on top of YOLOv5s-1, yielding a 2% improvement in mAP value compared to YOLOv5s-1. Finally, YOLOv5s-3 enhances the loss function based on YOLOv5s-2, utilizing the EIoU loss function, which leads to a 5% increase in mAP over that of YOLOv5s-2.

In summary, for the algorithm presented in this paper (YOLOv5s-3), which is a modification of the YOLOv5s algorithm, incorporating ShuffleNetv2 as a lightweight network structure, adding the ECA attention mechanism, and optimizing the loss function. These adjustments, when compared to the original YOLOv5s algorithm, enhance evaluation metrics such as algorithm precision and mAP. While the introduction of the lightweight network structure ShuffleNetv2 does result in a minor decrease in precision and recall rates, it substantially reduces algorithm parameters and computation requirements.

### Comparison of algorithms

This paper employs a two-stage detection algorithm to represent the Faster-RCNN model, while one-stage detection algorithm models including SSD, YOLOv3, YOLOv4, and YOLOv5s are selected for evaluating the proposed algorithm's effectiveness^58^. The experimental environment remains consistent across all algorithms, with training conducted over 300 rounds, and the experimental results are compared in Table 4.

**Table 4:** Comparison of experimental results of common algorithm models.

Algorithm model	mAP@0.5	Parameter volume/M	Computing volume/M
Faster-RCNN	0.841	137.06	186.32
SSD	0.631	27.32	32.28
YOLOv3	0.765	60.34	115.80
YOLOv4	0.798	64.36	100.23
YOLOv4-tiny	0.812	21.86	46.15
YOLOv5s	0.832	7.05	14.50
Ours	0.875	3.19	6.70

The experimental results above demonstrate that the algorithm proposed in this paper outperforms the others in terms of model size and detection precision.

When compared to the two-stage target detection algorithm, the algorithm in this paper exhibits a slightly smaller improvement in mAP value, but excels in both model size and detection speed. In comparison to other one-stage target detection algorithms, this algorithm performs exceptionally well in both speed and precision.

In summary, the algorithm presented in this paper achieves a 4.3% increase in mAP value, while simultaneously reducing parameter volume by 54.8% and computational workload by 53.8%. This configuration strikes a balance between light computational load and precision. Although the introduction of three modules results in a minor reduction in network processing speed, it still satisfies the requirement for real-time detection.

### Algorithm verification

**Figure 4:**
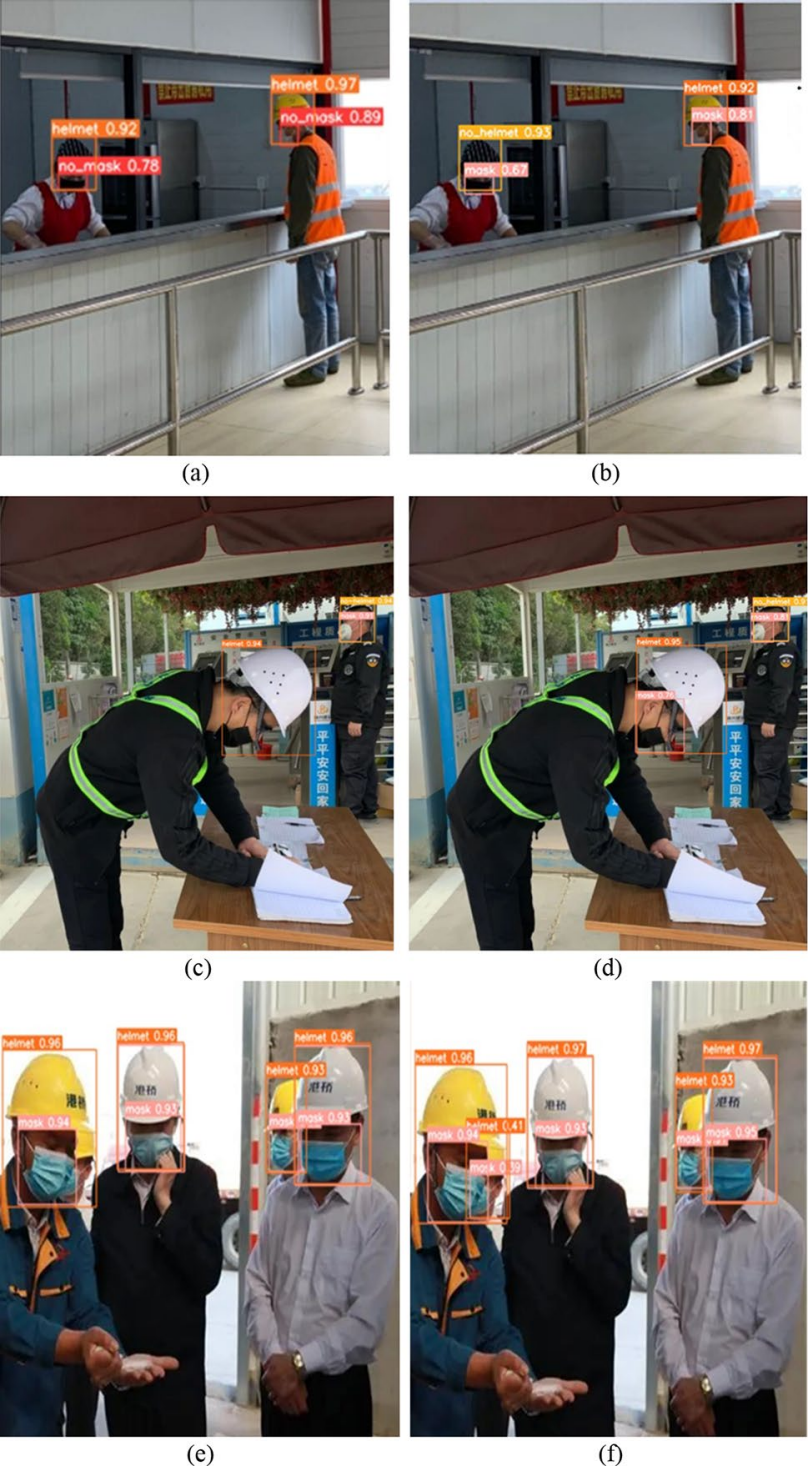
Detection results of the original YOLOv5 model and the improved YOLOv5 model.

This paper validates the algorithm's feasibility by conducting image detection under identical experimental conditions. The original YOLOv5s algorithm model and the optimized, enhanced model presented in this paper are tested and compared individually. The detection results are depicted in Fig. 4.

In the figures above, figures (a), (c), and (e) depict the detection results obtained using the original YOLOv5s algorithm, while figures (b), (d), and (f) showcase the outcomes achieved with the optimized algorithm proposed in this paper. Here's what the figures reveal:

Figures (a) and (b) depict indoor personnel detection in a canteen. The original YOLOv5s algorithm exhibits three false positive detections. Specifically, it fails to determine if individuals in the pictures are wearing masks, and it mistakenly identifies a person wearing a hat as wearing a helmet. In contrast, the improved algorithm correctly identifies these aspects.

Figures (c) and (d) show outdoor target detection. The original algorithm experiences instances of missed detection, particularly failing to identify whether a person who is lowering their head (possibly for recording) is wearing a mask. In contrast, the algorithm presented in this paper accurately identifies these cases, displaying higher confidence levels compared to the original algorithm.

Figures (e) and (f) in the figures reveal missed detections in the original algorithm for dense crowd scenarios. Conversely, the algorithm proposed in this paper comprehensively and accurately identifies targets in such conditions. Consequently, the improved algorithm demonstrates superior performance in both indoor and outdoor target detection scenarios.

## Conclusions

This paper presents a lightweight target detection algorithm, building upon YOLOv5s by enhancing the backbone network, incorporating an attention module, and optimizing the loss function to improve model detection precision. This approach yields significant improvements in both load and precision of the model. Experimental results demonstrate the algorithm's strong detection performance, effectively balancing precision and real-time capabilities. The average target detection precision, measured by mAP, is enhanced by 4.3% to reach 0.875. Furthermore, the model significantly reduces parameter volume and computing requirements by 54.8% and 53.8%, respectively, resulting in shorter detection times and improved precision. This model effectively meets the demand for a balanced solution between light resource utilization and precision, making it suitable for municipal project construction. While the improved model maintains real-time detection capabilities, there is a slight reduction in detection speed. Future research will focus on further optimizing detection speed and deploying the model on terminal devices, thus enhancing its applicability in real-world scenarios.

### Future outlook

Despite achieving some progress in simultaneous detection of safety helmets and masks, the authors acknowledge there is still room for improvement in this study. The focus of the research is solely on the detection methods of wearing safety protective equipment. In practical applications, it is necessary to establish a standardized system that includes an interaction interface between the backend algorithm and the frontend system. Such a system will enable real-time monitoring of construction sites and ultimately establish a comprehensive solution. In future work, the authors plan to continue enhancing detection accuracy and exploring more effective algorithms and technologies. Additionally, the authors intend to design and develop a system to ensure the compatibility of the algorithm with real-world application environments. By simultaneously considering the requirements of accuracy and real-time performance, our goal is to provide a high-performance, reliable system that offers a feasible and effective solution for detecting the simultaneous wearing of safety helmets and masks in municipal construction projects.

## Data Availability

Data are not publicly available and can be obtained by contacting the corresponding author if necessary.
